# Prevalence and characteristics of apical aneurysm in hypertrophic cardiomyopathy: a prospective analysis of cardiac magnetic resonance findings and clinical outcomes

**DOI:** 10.1186/1532-429X-17-S1-P278

**Published:** 2015-02-03

**Authors:** Sang-Chol Lee, Eun Kyoung Kim, Sung-A Chang, Sung-Ji Park, Yeon Hyeon Choe, Sung Mok Kim, Seung Woo Park, Jae K Oh

**Affiliations:** Dept. of cardiovascular Meidicine, Samsung Medical Center / Sungkyunkwan Unicersity, Seoul, Korea (the Republic of; Radiolgy, Samsung Medical Center / Sungkyunkwan University, Seoul, Korea (the Republic of; Cardiovascular Medicine, Mayo Clinic, Rochester, MN USA

## Background

Hypertrophic cardiomyopathy (HCM) with apical aneurysm (AAn) is associated with considerable morbidity and mortality. However, the real incidence of AAn tends to be underrecognized due to the poor visualization of left ventricular (LV) apex with echocardiography. This study sought to investigate the exact incidence and clinical manifestations of AAn in patients with HCM.

## Methods

A total of 350 consecutive patients diagnosed with HCM (mean age 54 ± 12, 278 males) underwent cardiac magnetic resonance (CMR) and echocardiography. All enrolled patients were prospectively followed up for adverse clinical events including cardiac death, admission for heart failure and cerebrovascular accident. We divided the subjects into 4 phenotypes according to the location of hypertrophic segment; asymmetrical septal hypertrophy (ASH), apical, concentric and septal/apical type. On CMR, the LV volumetric parameters were measured, and the amount of late gadolinium enhancement (LGE) was calculated with gray-scale thresholds of 6 SD above the mean signal intensity for normal remote myocardium. Echocardiographic evaluations included left atrial volume index, mitral inflow pattern, tissue Doppler of mitral annulus and LV dimension. Median follow up duration was 37 months.

## Results

The prevalence of AAn on CMR was 14.3%, which was significantly higher compared to previously reported data. AAn was detected in all groups of HCM regardless of type (16.8% in ASH type, 15.3% in apical type, 17.9% in concentric type, and 9.1% in septal/apical type of HCM). Clinical manifestations and LV volumetric parameters on CMR did not differ between the HCM patients with and without AAn. The frequency and the amount of LGE were not different between two groups (frequency; 94% vs. 93.3%, p=1.00, extent; 11.7±8.9 vs. 13.0±10.3, p=0.43). During follow up, the frequency of adverse clinical events did not differ between patients with and without AAn (p=0.259).

## Conclusions

The incidence of AAn in HCM patients was far higher than it was reported previously. Regardless of presence of AAn, initial manifestations and associated morphology of LV were similar. This means that adverse clinical outcomes in HCM patients with AAn may be a long-range problem which arises from secondary myocardial changes due to AAn.

